# Comparison of 6-min walk test distance vs*.* estimated maximum oxygen consumption for predicting postoperative pulmonary complications in patients undergoing upper abdominal surgery: a prospective cohort study

**DOI:** 10.1186/s13741-023-00309-z

**Published:** 2023-05-23

**Authors:** Xin Yin, Jingwen Wang, Zhibo Xu, Fuyong Qian, Songbin Liu, Yuxi Cai, Zhaoshun Jiang, Xixue Zhang, Weidong Gu

**Affiliations:** 1 Department of Anesthesiology, Huadong Hospital Affiliated to Fudan University, No 221, West Yan’an Road, Shanghai, 200040 China; 2 Department of Oncology, Huadong Hospital Affiliated to Fudan University, Shanghai, China

**Keywords:** 6-min walk test, Cardiorespiratory fitness, Pulmonary complication, Prediction, Surgery

## Abstract

**Objective:**

The present study aims to evaluate the predictive ability of estimated maximum oxygen consumption (e$\dot{V}$O_2max_) and 6-min walk distance (6MWD) for postoperative pulmonary complications (PPCs) in adult surgical patients undergoing major upper abdominal surgery.

**Method:**

This study was conducted by collecting data prospectively from a single center. The two predictive variables in the study were defined as 6MWD and e$\dot{V}$O_2max_. Patients scheduled for elective major upper abdominal surgery from March 2019 to May 2021 were included. The 6MWD was measured for all patients before surgery. e$\dot{V}$O_2max_ was calculated using the regression model of Burr, which uses 6MWD, age, gender, weight, and resting heart rate (HR) to predict aerobic fitness. The patients were categorized into PPC and non-PPC group. The sensitivity, specificity, and optimum cutoff values for 6MWD and e$\dot{V}$O_2max_ were calculated to predict PPCs. The area under the receiver operating characteristic curve (AUC) of 6MWD or e$\dot{V}$O_2max_ was constructed and compared using the *Z* test. The primary outcome measure was the AUC of 6MWD and e$\dot{V}$O_2max_ in predicting PPCs. In addition, the net reclassification index (NRI) was calculated to assess ability of e$\dot{V}$O_2max_ compared with 6MWT in predicting PPCs.

**Results:**

A total of 308 patients were included 71/308 developed PPCs. Patients unable to complete the 6-min walk test (6MWT) due to contraindications or restrictions, or those taking beta-blockers, were excluded. The optimum cutoff point for 6MWD in predicting PPCs was 372.5 m with a sensitivity of 63.4% and specificity of 79.3%. The optimum cutoff point for e$\dot{V}$O_2max_ was 30.8 ml/kg/min with a sensitivity of 91.6% and specificity of 79.3%. The AUC for 6MWD in predicting PPCs was 0.758 (95% confidence interval (CI): 0.694–0.822), and the AUC for e$\dot{V}$O_2max_ was 0.912 (95%CI: 0.875–0.949). A significantly increased AUC was observed in e$\dot{V}$O_2max_ compared to 6MWD in predicting PPCs (*P* < 0.001, *Z* = 4.713). And compared with 6MWT, the NRI of e$\dot{V}$O_2max_ was 0.272 (95%CI: 0.130, 0.406).

**Conclusion:**

The results suggested that e$\dot{V}$O_2max_ calculated from the 6MWT is a better predictor of PPCs than 6MWD in patients undergoing upper abdominal surgery and can be used as a tool to screen patients at risk of PPCs.

**Supplementary Information:**

The online version contains supplementary material available at 10.1186/s13741-023-00309-z.

## Background

Postoperative pulmonary complications (PPCs) after upper abdominal surgery are common, with a high incidence rate of 17–27.5% (Futier et al. 2013; Sevransky et al. 2008; Pasquina et al. 2006). PPCs have shown negative impacts on patient outcomes, including increased mortality and morbidity, prolonged hospital length of stay, and increased health care costs (LAS VEGAS investigators 2017; Miskovic and Lumb 2017; Serejo et al. 2007). Therefore, the preoperative prediction of PPCs and identification of high-risk patients undergoing upper abdominal surgery may contribute to early prevention and interventions such as smoking cessation, bronchodilator treatment, and/or respiratory muscle training, as well as intraoperative lung-protective ventilation strategies and effective postoperative pain management, and enhance postoperative recovery (Shander et al. 2011; Nijbroek et al. 2019).

Maximum oxygen consumption (VO_2max_) measured by cardiopulmonary exercise testing (CPET) can objectively reflect functional capacity. It is expressed in liters per minute with weight indexed values (ml/kg/min) (Snowden et al. 2010). VO_2 max_ can be used to assess exercise tolerance, and it is a valuable index for predicting the outcomes and complications of surgeries (Smith et al. 2013; Lee et al. 2006; Barakat et al. 2015). Currently, VO_2max_ is a reliable predictor of PPCs recommended by guidelines for thoracic surgery (Brunelli et al. 2013). However, CPET is a time-consuming and expensive. It requires specialized equipment and trained personnel (Krüger et al. 2006). The 6MWT is a simple, inexpensive alternative to CPET for assessing sub-maximal functional capacity in various patient populations due to its convenience and accessibility (Sinclair et al. 2012; Holland et al. 2014; Du et al. 2017; Singh et al. 2014). In recent years, 6MWT has been utilized in several clinical studies to predict postoperative complications, including PPCs (Keeratichananont et al. 2016; Ramos et al. 2021). The predictive validity of the 6MWD for PPCs has been reported to be poor due to its low sensitivity (Hattori et al. 2018; Paisani et al. 2012; Marjanski et al. 2015). Therefore, the method for PPCs prediction needs further improvement.

Burr et al. proposed an equation to calculate the estimated VO_2max_ (e$\dot{V}$O_2max_), which incorporated 6MWD, age, gender, body weight, and resting heart rate (RHR) (Burr et al. 2011). A strong correlation was established between e$\dot{V}$O_2max_ and VO_2max_ measured by CPET (Deka et al. 2021). e$\dot{V}$O_2max_ markedly improves the predictive capacity of aerobic fitness compared to 6MWD (Burr et al. 2011). However, the performance of e$\dot{V}$O_2max_ in the prediction of PPCs is yet unclear. In the present study, we tested the hypothesis that e$\dot{V}$O_2max_ had better predictive validity for PPCs than 6MWD in patients undergoing elective major upper abdominal surgery.

## Methods

### Participants

The present study is a prospective diagnostic study was conducted in a single center from March 2019–May 2021 at Huadong Hospital Affiliated with Fudan University, Shanghai, China. The inclusion criteria were as follows: (i) Patients scheduled for elective major upper abdominal surgery with an expected operation duration of at least 2 h and (ii) age ≥ 18 years. The main exclusion criteria (ATS statement 2002; Hammal et al. 2017) were as follows: (i) failure to perform 6MWT due to the limitation of movement or complications, (ii) current treatment with β-blockers, (iii) unstable angina or myocardial infarction during the previous month, (iv) systolic blood pressure > 180 mmHg or diastolic blood pressure > 120 mmHg or heart rate > 120 beats/min at rest, and (v) inability to cooperate with 6MWT because of communication disorders or mental disease. All patients provided written informed consent before enrolment in the study. This clinical study was conducted in accordance with the principles of the Declaration of Helsinki and approved by the ethics committee at Huadong Hospital (2019K015). The study was registered with the Chinese Clinical Trial Registry (ChiCTR1900022772).

### Data collection

Demographic data, including age, gender, and resting HR, were collected. 6MWT was performed 1 day before the operation. The operation duration, intraoperative blood loss and transfusion volume, and urine volume were recorded during the surgery.

For all patients, 6MWT was performed according to the American Thoracic Society guidelines by the same technician in the same time frame (13:00–15:00) 1 day before the surgery. After receiving standardized guidance, the patient walked back and forth along a long, flat, straight, enclosed 30-m corridor. Then, the 6MWD was calculated. Each patient was tested twice at an interval of 1 h, and the test results of a longer walking distance were recorded for data analysis. Before and after 6MWT, the Borg’s scale was used to measure the patients’ perceived level of effort or fatigue (ATS statement 2002).

### Predictive variables

Both 6MWD and e$\dot{V}$O_2max_ were measures used in this study to predict the incidence of PPCs. As described above, 6MWD is a measure of physical fitness that reflects a patient’s functional capacity. It is obtained through a simple and inexpensive 6MWT. e$\dot{V}$O_2max_ calculated by incorporating 6MWD, weight, gender, and resting HR was used to estimate the highest rate of oxygen during exercise.

### Calculation of e$\dot{V}$O_2max_

The e$\dot{V}$O_2max_ was calculated using the equation proposed by Burr et al. (2011).

$$e\dot V\;{\mathrm O}_{2\max}(\mathrm{ml}/\mathrm{kg}/\min)=70.161+(0.023\times6\mathrm{MWD})-(0.276\times\mathrm{weight})-(6.79\times\mathrm{gender},\;\mathrm{where}\;\mathrm{male}=0,\;\mathrm{female}=1)-(0.193\times\mathrm{RHR})-(0.191\times\mathrm{age})$$

### Outcomes

The primary outcome of this study was the predictive ability of e$\dot{V}$O_2max_ and 6MWD, as measured by the AUC, in predicting the incidence of PPCs within the first 7 postoperative days. The diagnostic criteria for PPCs described in previous studies (Kroenke et al. 1992; Hulzebos et al. 2006; Katsura et al. 2015) (Additional file 1) were applied in the present study. The PPCs were classified into grades 0–4 from mild to severe, based on clinical manifestations. Clinically significant PPCs was defined as two or more items in grade 2 complications or one item in grade 3 or 4 complications described previously (Hulzebos et al. 2006). The PPCs on days 2, 4, and 7 after the surgery were evaluated by independent researchers blinded to the value of 6MWD. The secondary outcomes were intensive care unit (ICU) admission rate, length of ICU stay, and postoperative 30-day mortality.

### Statistical analysis

The sample size was calculated using PASS version 15.0 (NCSS LLC, Kaysville, Utah, USA). A pilot study was conducted with 24 patients, in which the incidence of PPCs was 25%. The results of this pilot study showed that the area under the curve (AUC) for predicting PPCs was 0.718 (95%CI: 0.499–0.880) for 6MWD and 0.861 (95%CI: 0.659–0.967) for e$\dot{V}$O_2max_. The correlation between the two diagnostic tests is assumed to be 0.732 for the PPC group and 0.644 for the non-PPC group, respectively. Thus, a sample size of at least 171 cases was required (43 cases in the PPCs group), based on the expected incidence from the pilot study, assuming *α* = 0.05, 90% power of detection, and 20% shedding rate.

The data were presented as mean ± standard deviation or median (interquartile range). The enumeration data were presented in numbers and percentages. The baseline characteristics and postoperative outcomes of patients were shown by descriptive statistics. The normality and homogeneity of continuous variables were assessed using the Kolmogorov–Smirnov test and Levene test. Two independent samples *t* test or Mann–Whitney *U* test was used to compare the PPCs and the non-PPCs group. *χ*^2^ or Fisher’s exact probability test was used for the intergroup comparison of binary categorical variables. Pearson’s r correlations with 6MWD and e$\dot{V}$O_2max_ were calculated. To handle this missing data, we used the average value of similar cases to imputed (PPC patients or non-PPC patients). The predictive validity of the two methods was compared with respect to the sensitivity and specificity and analyzed by the receiver operating characteristic (ROC) curves. The AUC of the two methods was obtained and compared by *Z* test. Net reclassification index was calculated using the package “nricens” in the R statistical computing language (version 4.2.2; R Foundation for Statistical Computing; Auckland University, Auckland, New Zealand). All analyses were carried out using SPSS version 24.0 (IBM Corporation, NY, USA), and the data were considered statistically significant if the *P* value was < 0.05.

## Results

### Participant characteristics

**Fig. 1:**
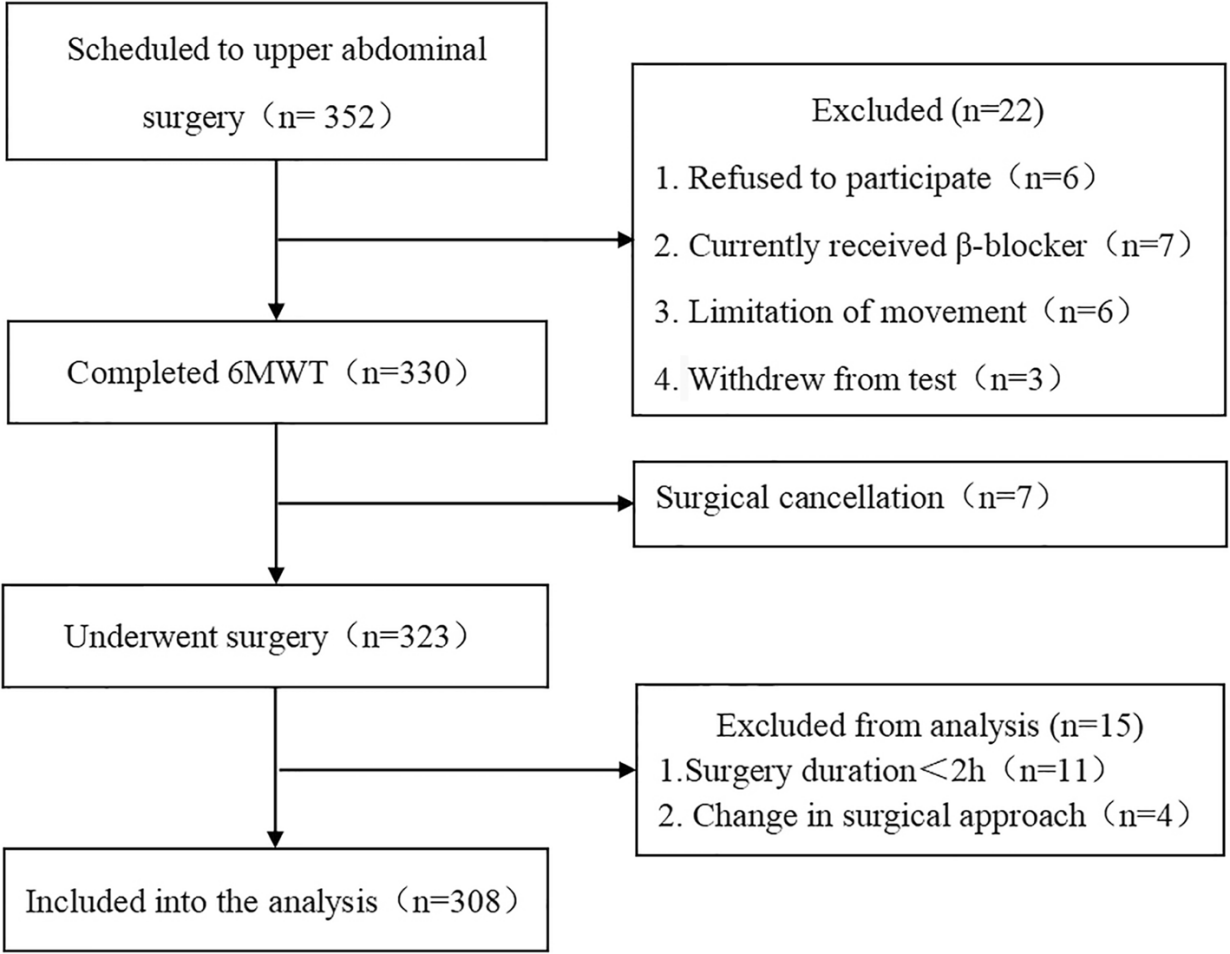
Flowchart of study participants. Abbreviations: 6MWT, 6-min walk test

Of the 352 patients scheduled for major upper abdominal surgery, 308 were included in the present study (Fig. 1). 71/308 (23.1%) patients developed clinically significant PPCs. The baseline values for demographic, preoperative, intraoperative, and postoperative observation items are listed in Table 1. Body mass index (BMI), age, resting HR, the ratio of hypertension and allergies, Forced Expiratory Volume in the first second/Forced Vital Capacity (FEV_1_/FVC), and blood loss were significantly higher in the PPC group than in the non-PPC group (*P* < 0.05).

**Table 1:** Characteristics of the patients

Characteristics	PPCs group (*n* = 71)	Non-PPC group (*n* = 237)	*P value*
Age (years)	73.0 (67.0–80.0)	64.0 (55.5–71.0)	< 0.0001
Gender			< 0.0001
Male	28 (39.4%)	152 (64.1%)	
Female	43 (60.6%)	85 (35.9%)	
Weight (kg)	63.0 (54.5–71.0)	62.0 (55.0–69.0)	0.615
BMI (kg/m^2^)	24.3 (21.6–26.2)	22.8 (20.3–24.6)	0.001
Waistline (cm)	90.0 (82.0–98.0)	84.0 (77.0–90.0)	< 0.0001
Smoking status			0.003
Current	11 (15.5%)	80 (33.8%)	
Never	60 (84.5%)	157 (66.2%)	
Comorbidities			
Hypertension	43 (60.6%)	90 (38.0%)	0.001
Diabetes	12 (16.9%)	38 (16.0%)	0.862
Thyroid disease	6 (8.5%)	15 (6.3%)	0.534
Cancer	65 (91.5%)	181 (76.4%)	0.005
Pulmonary disease	10 (14.1%)	18 (7.6%)	0.095
Allergies	15 (21.1%)	20 (8.4%)	0.003
Respiration rate (breaths/min)	17 (15–19)	16 (15–18)	0.119
Systolic BP (mmHg)	130 (120–136)	124.0 (118.5–134.0)	0.007
Diastolic BP (mmHg)	70 (64–80)	73.1 (68–80)	0.295
Resting heart rate (bpm)	81 (73–95)	71 (66–81)	0.000
FEV_1_/FVC (%)	88.1 (81.2–92.2)	91.3 (85.3–95.5)	0.005
Surgery duration (min)	247 (163–310)	211 (170–280)	0.274
Surgical technique			0.139
Open abdominal	50 (70.4%)	144 (60.8%)	
Laparoscopic	21 (29.6%)	93 (39.2%)	
Surgery type			0.184
Pancreatectomy	21 (29.6%)	54 (22.8%)	
Hepatectomy	3 (4.2%)	27 (11.4%)	
Gastrectomy	33 (46.5%)	125 (52.7%)	
Splenectomy	4 (5.6%)	7 (3.0%)	
Transverse colectomy	10 (14.1%)	24 (10.1%)	
Blood loss (mL)	210 (120–400)	200 (135–390)	0.263
Fluid infusion (mL)	2600 (2000–3100)	2500 (2000–3100)	0.657
Blood transfusion (mL)^a^	0 (0–1500)	0 (0–1900)	0.059
Blood transfusion	8 (11.3%)	12(5.1%)	0.094
Urine (mL)	610 (395–800)	500 (300–800)	0.186
Rate of ICU admission (%)	33.8	6.8	< 0.0001
Length of ICU stay (day)	0 (0–2)	0 (0–0)	< 0.0001

Mean ± standard deviation (SD), median (interquartile range), number (%)

*Abbreviations: PPCs* postoperative pulmonary complications, *BMI* body mass index, *BP* blood pressure, *FEV*_*1*_ forced expiratory volume in the first second of expiration, *FVC* forced vital capacity, *ICU* intensive care unit

^a^Expressed as median (min–max)

### 6MWD and e$\dot{V}$O_2max_

No significant difference was detected in the Borg score between the two groups before and after 6MWT. Both tests showed that < 5% of patients presented moderate shortness of breath or fatigue post-test (Additional file 2).

**Fig. 2:**
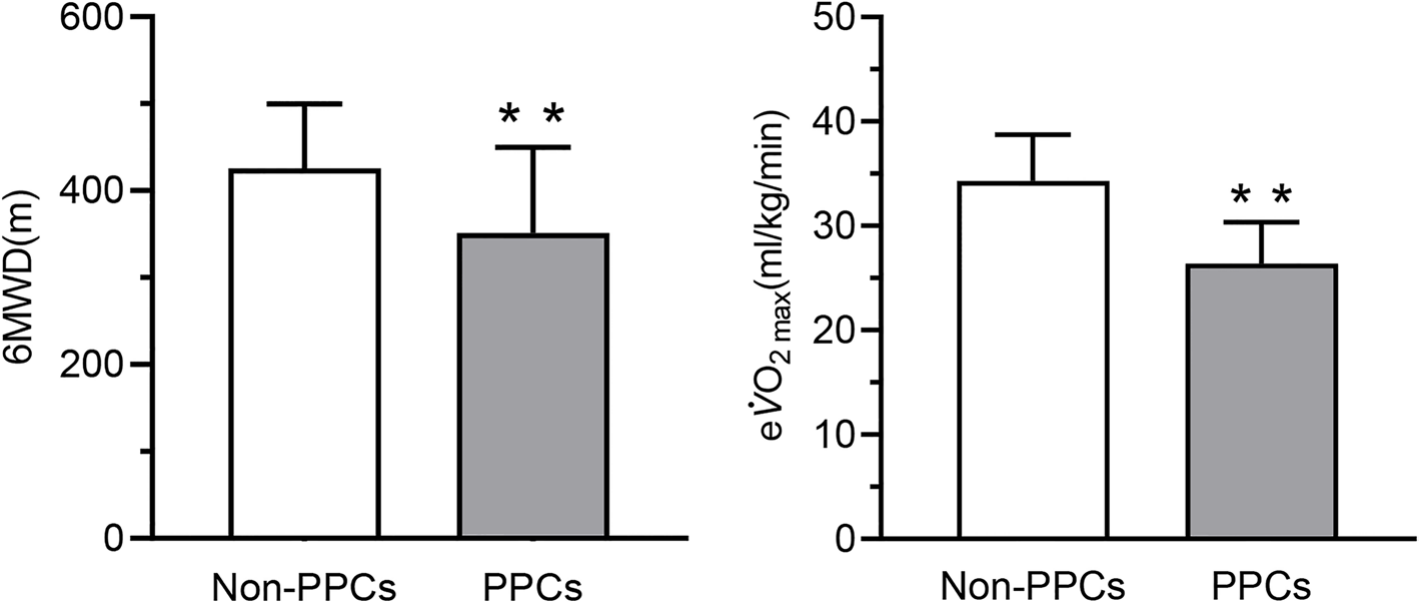
Comparison of 6MWT distance and eVO_2max_ between the two groups. Abbreviations: 6MWD, 6-min walk test distance; eVO_2max_, estimated maximum oxygen consumption; PPCs, postoperative pulmonary complications

Both 6MWD (342.8 ± 90.9 m *vs*. 425.6 ± 74.1 m, *P* < 0.001) and e$\dot{V}$O_2max_ (26.4 ± 4.0 ml/kg/min vs. 34.3 ± 4.4 ml/kg/min, *P* < 0.001) were significantly lower in the PPC group than in the non-PPC group (Fig. 2).

### Comparison of 6MWD and e$\dot{V}$O_2max_ for predicting PPCs

**Fig. 3:**
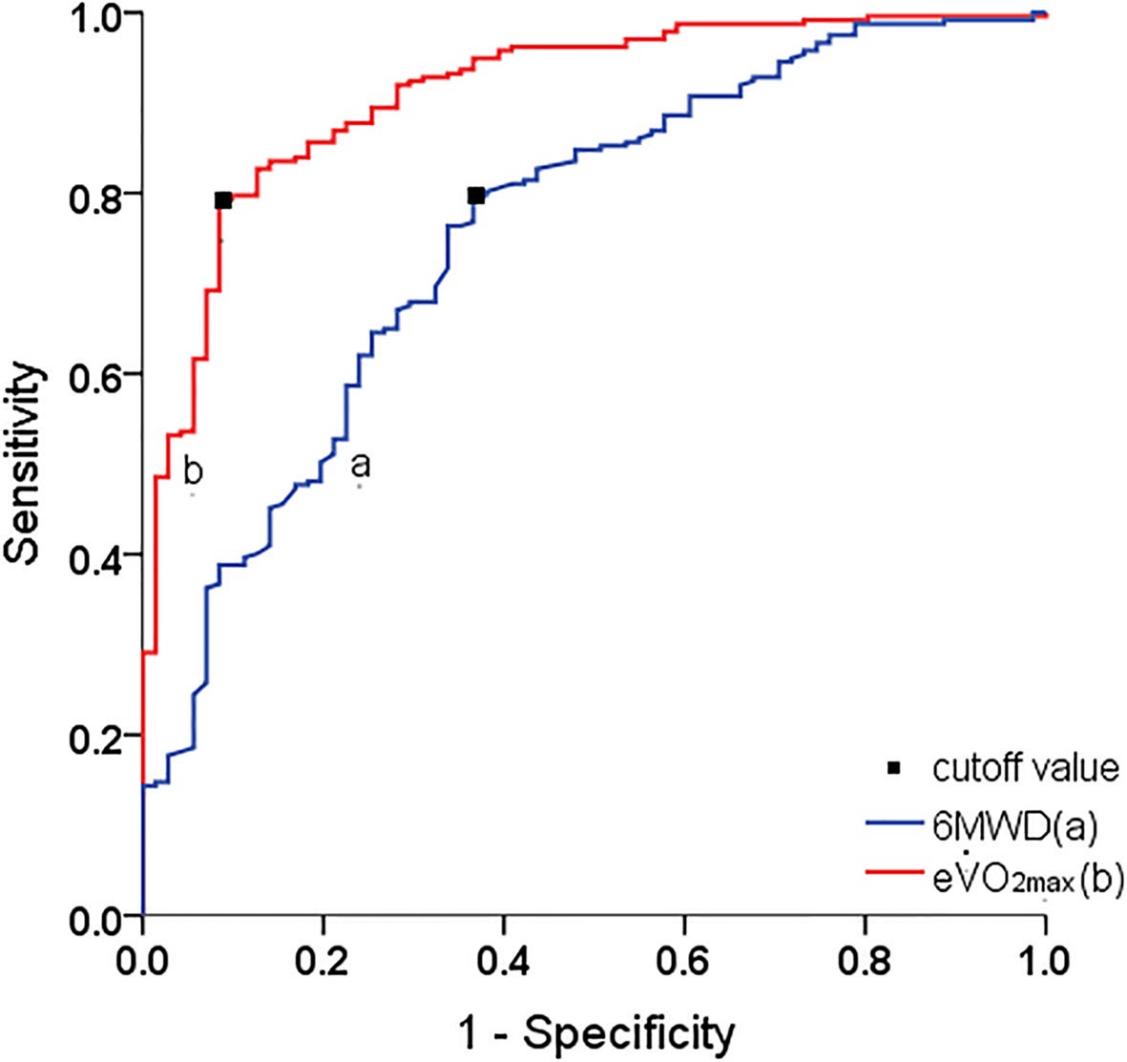
ROC curve for 6MWD (blue) and e$\dot{V}$O_2max_ (red) in the prediction of PPCs, with cutoff value indicated by black squares. Abbreviations: 6MWD, 6-min walk test distance; e$\dot{V}$O_2max_, estimated maximum oxygen consumption

The optimum cutoff for 6MWD in predicting PPCs was 372.5 m with a sensitivity of 63.4% and specificity of 79.3% (AUC 0.758, 95%CI: 0.694–0.822). The optimum cutoff for e$\dot{V}$O_2max_ was 30.8 ml/kg/min with a sensitivity of 91.6% and specificity of 79.3% (AUC 0.912, 95%CI: 0.875–0.949). Significantly increased AUC was observed in e$\dot{V}$O_2max_ compared to 6MWD (*P* < 0.001, *Z* = 4.713) (Fig. 3 and Table 2). The results showed that the ability of e$\dot{V}$O_2max_ for predicting PPCs was consistently higher than that of 6MWD on days 2, 4, and 7, as measured by AUC (Additional file 3). Furthermore, NRI was calculated to evaluate the predictive improvement of e$\dot{V}$O_2max_ using point estimates with a cutoff value of 372.5 m for 6WMT and 30.8 ml/kg/min for e$\dot{V}$O_2max_. The NRI was 0.272 (95%CI: 0.130, 0.406), indicating that the use of e$\dot{V}$O_2max_ as a predictor improved the classification of PPCs over 6WMT at these cutoff values.

**Table 2:** Characteristics of the ROC curves

	6MWD (*n* = 308)	e$\dot{V}$O_2max_ (*n* = 308)
Cutoff values	372.5 (m)	30.8 (ml/kg/min)
AUC	0.758	0.912
Sensitivity (%)	63.4	91.6
Specificity (%)	79.3	79.3
Positive predictive value (%)	47.9	57.0
Negative predictive value (%)	87.9	96.9

### ICU admission rate, LOS, and mortality within 30 days after the operation

40/308 (13.0%) patients were admitted to the ICU after the operation. The ICU admission rate of the PPC group was higher than the non-PPC group (33.8 vs. 6.8%, *P* < 0.001). The median LOS of all patients in the ICU was 0 (0–0) days. Patients with PPCs experienced longer LOS than those without PPCs (0 (0–2) days vs. 0 (0–0) days,* P* < 0.001). The follow-up was conducted via telephone at 30 days postoperatively; 14/308 patients were lost to follow-up, and 11/294 (3.7%) patients were deceased. The mortality between the PPC and the non-PPC group differed significantly (11.8 vs. 1.3%, *P* < 0.001).

## Discussion

The results showed that e$\dot{V}$O_2max_ is a better predictor of PPCs than 6MWD in patients undergoing major upper abdominal surgery. Herein, we used the diagnostic criteria of PPCs proposed by Kroenke et al., including the symptoms, signs, imaging examination, biological detection, and treatment. The diagnostic criteria have been applied as they are well-established and easy to use in clinical settings (Kroenke et al. 1992; Hulzebos et al. 2006; Futier et al. 2013; Katsura et al. 2015; Costa Leme et al. 2017). The incidence of PPCs was 23.1% in patients undergoing major upper abdominal surgery, which was consistent with the incidence of 27.5% in a previous study (Futier et al. 2013).

6MWT is a less resource-intensive method that reflects the submaximal functional capacity of a patient through walking distance. It can be used to confirm a patient’s functional status and ability of daily living (Faulkner et al. 2012; Miccichè et al. 2019; Studenski et al. 2011). 6MWT is a popular approach in the clinic to predict the risk of PPCs (Lee et al. 2020; Soares and Nucci 2021). However, the application of 6MWD has some limitations. In the study by Marjanski et al., the sensitivity of 6MWD to predict PPCs in patients undergoing lobectomy was only 36% (Marjanski et al. 2015). In a recent review, Moran et al. reported that 6MWD might be suitable for predicting the general complications rather than cardiac or pulmonary complications (Moran et al. 2016). The results of the present study suggested that the optimal cutoff for 6MWD in predicting PPCs was 372.5 m with a sensitivity of only 63.4% and specificity of 79.3%. The present findings were in line with the previous studies in that the optimal predictive validity of 6MWD in PPCs was not determined yet.

VO_2max_ assessed by CPET can accurately reflect individual cardiorespiratory fitness, a prognostic indicator for surgery patients (Church et al. 2001; Smith et al. 2013). It also predicts postoperative complications sensitively and reliably (Benzo and Sciurba 2010; Win et al. 2005). Despite high accuracy of CPET, the complexity and high cost may limit its use. In contrast, e$\dot{V}$O_2max_ has the advantage of simplicity and ease of access, making it potentially more widely applicable.

Compared to 6MWD, e$\dot{V}$O_2max_ derived from 6MWD in combination with age, gender, body weight, and resting HR has a stronger correlation with VO_2max_ (Burr et al. 2011; Ross et al. 2010). Recently, e$\dot{V}$O_2max_ calculated by the Burr equation has been proved to be an effective and reliable assessment of functional capacity (Sitlinger et al. 2021; Yau et al. 2017). The present study showed that the predictive sensitivity of e$\dot{V}$O_2max_ for PPCs was 91.6%, and the specificity was 79.3% (AUC 0.912, 95%CI: 0.875–0.949). The comparison of ROC curves indicated that the AUC of e$\dot{V}$O_2max_ was significantly greater than that of 6MWD. In addition, the NRI for e$\dot{V}$O_2max_ was 0.272 (95%CI: 0.130, 0.406), indicating that it had a superior ability to reclassify individuals into more accurate risk categories compared to 6MWD. These results suggest that e$\dot{V}$O_2max_ has a superior ability to predict PPCs compared to 6MWD alone. This might be because the Burr equation also incorporates additional age, gender, body weight, and resting HR in addition to 6MWD, which offer a more comprehensive representation of the patient’s functional capacity. Previous studies have shown that age, gender, and weight were independent risk factors for PPCs (Miskovic and Lumb 2017). According to McAlister et al., age, surgery duration, and indwelling gastric tube were independent predictors of PPCs in non-thoracic surgery patients (McAlister et al. 2005). Hayashi et al. advocated that 6MWD, BMI, and intraoperative blood loss were independent risk factors for PPCs in pancreatic and liver surgery (Albouaini et al. 2007; Hayashi et al. 2017). These variables reflect the functional reserves of respiratory, circulatory, and metabolic systems under physiological load or stress and are closely related to the capacity of gas exchange, oxygen uptake, motor function of the skeletal muscle, and hemodynamic status. Notably, e$\dot{V}$O_2max_ has a high sensitivity (91.6%) in predicting PPCs, deeming it suitable for preoperative screening of patients with a high risk of PPCs undergoing major upper abdominal surgery.

Furthermore, the present study found that the ICU admission rate in the PPC group was significantly higher than in the non-PPC group. Also, significantly increased LOS and all-cause mortality within 30 days were observed in the PPC group compared to the non-PPC group. Aggravation or new postoperative complications caused by PPCs might lead to the abovementioned effects. These results suggested that to accelerate recovery and reduce medical burden, prevention, and treatment measures should be implemented in time for patients with a high risk of PPCs.

Nevertheless, the present study has several limitations. Firstly, for patients unable to walk or with 6MWT contraindications, e$\dot{V}$O_2max_ could not be calculated to predict PPCs, thereby necessitating further investigation in such a population. Secondly, this was a single-center study which may limit the generalizability of our findings.

In summary, e$\dot{V}$O_2max_ was superior to 6MWD in predicting PPCs in patients undergoing major upper abdominal surgery. The high sensitivity in predicting PPCs and simplicity of implementation indicated e$\dot{V}$O_2max_ as a promising preoperative screening tool.

## Supplementary Information

**Additional file 1.** Operational definitions of postoperative pulmonary complications.

**Additional file 2.** Borg scale before and after 6MWT.

**Additional file 3.** ROC curve characteristics of 6MWD and e$\dot{V}$O_2max_ on the 2nd, 4th, and 7th day.

## Data Availability

The data that support the findings of this study are available from the corresponding author upon reasonable request.

## Abbreviations

6MWD: 6-Min walk test distance
6MWT: 6-Min walk test
AUC: Area under the receiver operating characteristic curve
CPET: Cardiopulmonary exercise testing
e $\dot{V}$ O_2max_: Estimated the maximum oxygen consumption
FEV1/FVC: Forced expiratory volume in the first second/forced vital capacity
HR: Heart rate
NRI: Net reclassification index
PPCs: Postoperative pulmonary complications
ROC: Receiver operating characteristic
