# Supplementary material for: Comparison of 6-min walk test distance vs. estimated maximum oxygen consumption for predicting postoperative pulmonary complications in patients undergoing upper abdominal surgery: a prospective cohort study
Source: Perioper Med (Lond). 2023 May 23;12:18. doi: 10.1186/s13741-023-00309-z (PMC10207746; doi:10.1186/s13741-023-00309-z)
Supplement: Supplementary file 1 — Additional file 1. Operational definitions of postoperative pulmonary complications. [file 13741_2023_309_MOESM1_ESM.docx]

Additional file 1

| **Grade 1**  Cough, dry  Microatelectasis: abnormal lung findings and temperature >37.5 °C without other  documented cause; results of chest radiograph either normal or unavailable  Dyspnea, not due to other documented cause |
| --- |
| **Grade 2**  Cough, productive, not due to other documented cause  Bronchospasm: new wheezing or preexistent wheezing resulting in changes in the therapy  Hypoxemia: alveolar-arterial gradient >29 and symptoms of dyspnea or wheezing  Atelectasis: radiological confirmation plus either temperature >37.5 °C or abnormal lung findings  Hypercarbia, transient, and requiring treatment, such as naloxone or increased manual or mechanical ventilation  Adverse reaction to pulmonary medication |
| **Grade 3**  Pleural effusion, resulting in thoracentesis  Pneumonia, suspected: radiological evidence without bacteriological confirmation  Pneumonia, proved: radiological evidence and documentation of pathological organism by Gram-staining or culture  Pneumothorax  Postoperative reintubation or intubation, period of ventilator dependence does not exceed 48 hours |
| **Grade 4**  Ventilatory failure: postoperative ventilator dependence exceeding 48 hours or reintubation with subsequent period of ventilator dependence exceeding 48 hours |
| Source: Proposed by Kroenke et al. and revised by Hulzebos et al. |

Operational Definitions of Postoperative Pulmonary Complications
