# Supplementary material for: Comparison of 6-min walk test distance vs. estimated maximum oxygen consumption for predicting postoperative pulmonary complications in patients undergoing upper abdominal surgery: a prospective cohort study
Source: Perioper Med (Lond). 2023 May 23;12:18. doi: 10.1186/s13741-023-00309-z (PMC10207746; doi:10.1186/s13741-023-00309-z)
Supplement: Supplementary file 2 — Additional file 2. Borg scale before and after 6MWT. [file 13741_2023_309_MOESM2_ESM.docx]

Additional file 2

Borg scale before and after 6MWT [n (%)]

|  | Test 1 | | | | Test 2 | | | |
| --- | --- | --- | --- | --- | --- | --- | --- | --- |
|  | Shortness of breath | | Fatigue | | Shortness of breath | | Fatigue | |
|  | Pre-test | Post-test | Pre-test | Post-test | Pre-test | Post-test | Pre-test | Post-test |
| Nothing | 282(91.6) | 151(49.0) | 262(85.1) | 170(55.2) | 281(91.2) | 148(48.1) | 261((84.7) | 167(54.2) |
| Very slight | 17(5.5) | 64(20.8) | 27(8.8) | 50(16.2) | 17(5.5) | 66(21.4) | 28(9.1) | 51(16.6) |
| Very slight | 5(1.6) | 46(14.9) | 12(3.9) | 60(19.5) | 6(1.9) | 45(14.6) | 12(3.9) | 59(19.2) |
| Slight | 4(1.3) | 41(13.3) | 6(1.9) | 22(7.1) | 4(1.3) | 42(13.6) | 6(1.9) | 24(7.8) |
| Moderate | 0（0） | 5(1.6) | 1(0.3) | 5(1.6) | 0（0） | 6(1.9) | 1(0.3) | 6(1.9) |
| Somewhat severe | 0（0） | 1(0.3) | 0（0） | 1(0.3) | 0（0） | 1(0.3) | 0（0） | 1（0.3） |
| Severe | 0（0） | 0（0） | 0（0） | 0（0） | 0（0） | 0（0） | 0（0） | 0（0） |
| Very severe | 0（0） | 0（0） | 0（0） | 0（0） | 0（0） | 0（0） | 0（0） | 0（0） |
| Very very severe | 0（0） | 0（0） | 0（0） | 0（0） | 0（0） | 0（0） | 0（0） | 0（0） |
