# Supplementary material for: Comparison of 6-min walk test distance vs. estimated maximum oxygen consumption for predicting postoperative pulmonary complications in patients undergoing upper abdominal surgery: a prospective cohort study
Source: Perioper Med (Lond). 2023 May 23;12:18. doi: 10.1186/s13741-023-00309-z (PMC10207746; doi:10.1186/s13741-023-00309-z)
Supplement: Supplementary file 3 — Additional file 3. ROC curve characteristics of 6MWD and e$\dot{V}$V˙O2max on the 2nd, 4th, and 7th day. [file 13741_2023_309_MOESM3_ESM.docx]

Additional file 3
ROC curve Characteristics of 6MWD and e$\dot{V}$O_2max_ on the 2nd, 4th and 7th day

|  | Day2 | | Day4 | | Day7 | |
| --- | --- | --- | --- | --- | --- | --- |
|  | 6MWD | e$\dot{V}$O_2max_ | 6MWD | e$\dot{V}$O_2max_ | 6MWD | e$\dot{V}$O_2max_ |
| Patients (n) | 283 | 283 | 255 | 255 | 244 | 244 |
| Cutoff values | 382(m) | 30.8  (ml/kg/min) | 360(m) | 30.8 (ml/kg/min) | 285(m) | 29.5 (ml/kg/min) |
| AUC(95% CI) | 0.808 (0.757-0.852) | 0.921 (0.884-0.950) | 0.692 (0.631-0.748) | 0.889  (0.843 - 0.924) | 0.603 (0.538-0.665) | 0.910  (0.867 -0.943) |
| Sensitivity (%) | 73.9 | 91.3 | 55.6 | 88.9 | 28.6 | 100.0 |
| Specificity (%) | 76.4 | 79.3 | 82.7 | 79.7 | 96.6 | 85.7 |
| Positive predictive value (%) | 37.8 | 46.2 | 19.6 | 25.0 | 20.0 | 17.1 |
| Negative predictive value (%) | 93.8 | 97.9 | 96.1 | 99.0 | 97.9 | 100.0 |
| Z-test | 3.567 (*P*= 0.0004) | | 2.663 (*P*= 0.0077) | | 2.292 (*P*=0.0219) | |
